# Beyond “How are you?”

**DOI:** 10.1017/S1478951526102697

**Published:** 2026-05-13

**Authors:** Augustin Joseph

**Affiliations:** Palliative and Supportive Care Department, Mid-Atlantic Permanente Medical Group, Timonium, MD, USA

There’s a small moment at the start of every patient encounter that carries more weight than we often realize: how we introduce ourselves.

In the hospital, we are trained, implicitly or explicitly, to begin with “How are you?” It is automatic, polite, and deeply ingrained. It feels like the right thing to say. But for someone lying in a hospital bed, often in pain, uncertain, or frightened, the question can feel disconnected from reality. It asks for a socially acceptable answer at a moment when nothing feels socially acceptable. Many patients respond with “fine,” not because it is true, but because it is expected. In doing so, the conversation can begin with a kind of quiet misalignment.

That misalignment is subtle, but it matters. It sets a tone where the patient is asked, even unintentionally, to smooth over their experience. To make things easier. To meet us where we are, rather than the other way around. And once that tone is set, it can be difficult to move beyond it.

Independent of any formal institutional policy, I have adjusted my personal approach, moving away from that opening.

Instead, I introduce myself simply: “I’m Dr. Joseph. It’s a pleasure to meet you.” Then I pause. That pause is purposeful. It allows the patient to greet me in their own way and sets a tone of mutual respect rather than a transaction. In a setting where patients are often seen by many clinicians in quick succession, even a brief moment of stillness can signal that this interaction may be different.

I follow with orientation and acknowledgment: “I work with the other doctors taking care of you, and I’ve had a chance to read about what’s been going on.” In that sentence, I hope to convey 2 things. First, that I am part of their team, not an outsider. Second, that I have taken the time to understand their situation, at least in part, before walking into the room. Patients are often asked to retell their story again and again. Letting them know that I am already familiar with the outline can ease that burden.

More importantly, I try to name what is often unspoken: “It seems like you’ve been going through a lot over the past days and weeks.”

This is not a detailed summary of their medical course. It is a simple acknowledgment of difficulty. It does not require precision to be meaningful. Patients rarely correct this statement. More often, they nod, or pause, or take a breath. It creates a shared understanding that things are not okay, and that it is acceptable to say so.

Only then do I ask, “How are you holding up?”

It is a small shift in language, but a meaningful one. “How are you holding up?” invites honesty. It recognizes that the patient may be struggling and centers their experience rather than their condition. It opens the door to emotion, coping, and lived experience, instead of a reflexive, socially acceptable response.

Not every patient answers this question in the same way. Some speak openly about fear or frustration. Others give short answers. Some redirect to physical symptoms. But the question itself changes the range of what feels possible to say. It signals that I am not only interested in their disease, but in how they are experiencing it.

These first few sentences are not just pleasantries. They are clinical tools. They reduce confusion, demonstrate preparation, and acknowledge reality. They create space for the patient to be more than a diagnosis or a problem to be solved. They shape whether a patient feels seen as a person or managed as a task.

When we talk to patients, we spend a great deal of time preparing for difficult conversations. We learn frameworks, memorize phrases, and practice responding to emotion. But we give far less thought to how we begin.

How we introduce ourselves is not a trivial detail. It is the first signal of how we will show up.

And in many ways, it shapes everything that follows.

